# Geo-Distribution Patterns of Soil Fungal Community of *Pennisetum flaccidum* in Tibet

**DOI:** 10.3390/jof8111230

**Published:** 2022-11-21

**Authors:** Guangyu Zhang, Zhenxi Shen, Gang Fu

**Affiliations:** Lhasa Plateau Ecosystem Research Station, Key Laboratory of Ecosystem Network Observation and Modeling, Institute of Geographic Sciences and Natural Resources Research, Chinese Academy of Sciences, Beijing 100101, China

**Keywords:** biodiversity, climate change, elevation variation, latitude variation, longitude variation, multiple soil classes, Tibetan Plateau

## Abstract

*Pennisetum flaccidum* can be used as a pioneer species for the restoration of degraded grasslands and as a high-quality forage for local yak and sheep in alpine regions. The geographical distribution pattern of soil fungal community can modify that of *P. flaccidum*. A field survey along 32 sampling sites was conducted to explore the geo-distribution patterns of soil fungal community of *P. flaccidum* in Tibet. Soil fungal species, phylogenetic and function diversity generally had a closer correlation with longitude/elevation than latitude. The geo-distribution patterns of soil fungal species, phylogenetic and function diversity varied with soil depth. Soil fungal species, phylogenetic and function diversity had dissimilar geo-distribution patterns. Precipitation had stronger impacts on total abundance, species α-diversity, phylogenetic α-diversity, and function β-diversity than temperature for both topsoil (0–10 cm depth) and subtopsoil (10–20 cm depth). Furthermore, precipitation had stronger impacts on function α-diversity for topsoil, species β-diversity for topsoil, and phylogenetic β-diversity for subtopsoil than temperature. The combination of species, phylogenetic and function diversity can better reflect geo-distribution patterns of soil fungal community. Compared to global warming, the impact of precipitation change on the variation in soil fungal community of *P. flaccidum* should be given more attention.

## 1. Introduction

The Tibetan plateau is a vital alpine area that endures simultaneous changes from both climate change (e.g., global warming) and human activities (e.g., grazing) [1,2,3,4,5,6,7]. Due to the implementation of various ecological projects (e.g., grazing withdrawal project), and warmer and wetter climate conditions, alpine ecosystems overall have become better on the Qinghai-Tibet Plateau. However, alpine ecosystems tend to show varying degrees of degradation due to climatic changes such as increase in global temperature and low precipitation and overgrazing in some local areas [1,4,8,9,10]. The great theory that green mountains and water are equal to gold and silver mountains inspires us to restore these local degraded ecosystems. Natural restoration of degraded grasslands is an advocated, great scientific theory, and species selection is the key step for the restoration of degraded ecosystems. Native species with high stress resistance might be the best choice for species selection to achieve natural restoration of degraded ecosystems. On the other hand, along with the elimination of absolute poverty, the living standards of local residents have been gradually improved, and the demand for meat (e.g., yak meat) and milk by local community is rising on the Qinghai-Tibet Plateau. However, forage shortage is still a key bottleneck restricting the development of local animal husbandry on the Qinghai-Tibet Plateau. Planting local forage grass with high quality might be an important way to alleviate the contradiction between livestock and forage grass on the Qinghai-Tibet Plateau [3,4]. Thus, it is essential to select and promote the production of high-quality native forages and herbs on the Qinghai-Tibet Plateau.

*Pennisetum flaccidum* can be chosen as a pioneer species for the restoration of degraded grasslands and as a high-quality forage for local yak and sheep because of its developed rhizome systems, strong resistance to environmental stresses (e.g., dry, cold conditions), great nutritive value and wide geographical distribution on the Qinghai-Tibet Plateau [11]. Soil fungal community is an important bridge of nutrient elements (e.g., nitrogen and phosphorus) between plants and soils, and some fungal species might have favorable impacts on plants [12,13,14,15]. Soil fungal community can modify plant root activities and sequentially botany physiology and growth from various aspects [12,16]. The geographical distribution pattern of soil fungal community can modify that of host plant (e.g., *P. flaccidum*). However, to our knowledge, no reports have examined the geographical distribution pattern of soil fungal community associated with *P. flaccidum* on the Qinghai-Tibet Plateau. Consequently, we explored the geographical distribution pattern of soil fungal community of *P. flaccidum* and related driving mechanisms for their interaction in Tibet.

## 2. Materials and Methods

### 2.1. Study Area

**Figure A1:**
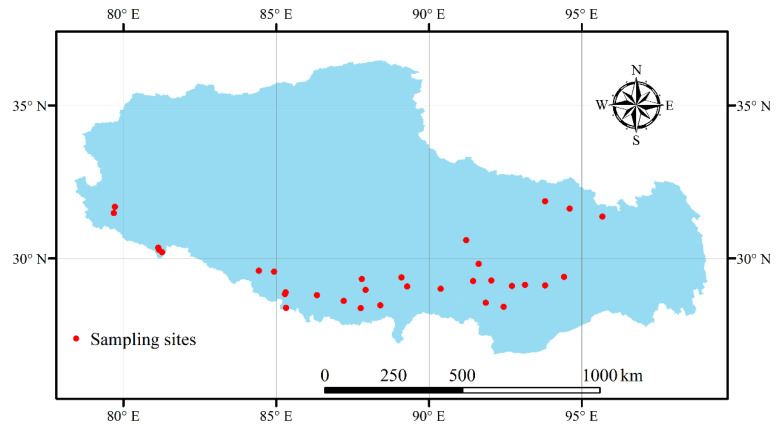
Location of sampling sites in Tibet.

Field samples were collected from 32 sampling sites in 2020 in Tibet (Figure A1 in Appendix A). Three geographic variables (i.e., latitude, longitude, elevation) and four climatic variables (MAP: mean annual precipitation in 1982–2020, MAT: mean annual temperature in 1982–2020, AP2020: annual precipitation in 2020, AT2020: annual temperature in 2020) were acquired for each one of the 32 sampling sites. Original monthly air temperature and precipitation were acquired from the China Meteorological Data Sharing Service System and later spatially interpolated to acquire the climate data for the whole area with a spatial resolution of 1 km × 1 km using the ANUSPLIN 4.3 software [3,4]. The four climatic variables were based on interpolated climate data. The elevation ranged from 2785 m to 4578 m. The longitude ranged from 79.69° E to 95.68° E. The latitude ranged from 28.37° N to 31.86° N. The MAT ranged from 2.33 °C to 13.02 °C. The MAP ranged from 73.70 mm to 640.16 mm. The AT2020 ranged from 2.91 °C to 12.25 °C. The AP2020 ranged from 26.88 mm to 785.87 mm for the 32 sampling sites.

### 2.2. Plant Sampling and Observation, Soil Sampling and Analyses

We observed stem diameter (SD), plant height and leaf area (LA). Subsequently, topsoil (0–10 cm depth) and subtopsoil (10–20 cm depth) were gathered at each one of the 32 sites. There were three replicates for each one of the 32 sites. Subsamples of these soils were stored in liquid nitrogen to observe soil fungal community. The PCR amplification primers for soil fungal community were BD-ITS1F (CTTGGTCATTTAGAGGAAGTAA) and ITS2-2043R (GCTGCGTTCTTCATCGATGC). The purified 50 ng DNA was mixed with 25 μL 2× Premix Taq, 1 μL Primer-F (10 μM) and 1 μL Primer-R (10 μM), and then mixture was determined to 50 μL by adding nuclease-free water. The PCR amplification included the following procedures (1) pre-degeneration for 5 min at 94 °C, (2) 30 cycles of denaturation at 94 °C for 30 s, annealing at 52 °C for 30 s, and extension at 72 °C for 30 s, (3) final elongation at 72 °C for 10 min. The PCR amplification instrument was the BioRad S1000 (Bio-Rad Laboratory, Hercules, CA, USA). The fragment length and concentration of the amplification PCR products were detected by 1% agarose gel electrophoresis. After the concentration of PCR products was compared by GeneTools Analysis Software (Version 4.03.05.0, SynGene), the PCR products were mixed according to the principle of equal mass. The PCR mixture was purified using the E.Z.N.A.^®^ Gel Extraction Kit (Omega, Norcross, GA, USA) gel recovery kit. The NEBNext^®^ Ultra™ II DNA Library Prep Kit for Illumina^®^ (New England Biolabs, Ipswich, MA, USA) was used for library construction. The Illumina Nova 6000 platform was used for PE250 sequencing (Guangdong Magigene Biotechnology Co., Ltd. Guangzhou, China). The double-ended raw reads data were clipped by sliding window quality (-W 4-M 20) on the fastp tool (an ultra-fast all-in-one FASTQ preprocessor, version 0.14.1, https://github.com/OpenGene/fastp, accessed on 30 September 2022). According to the sequence at the ends of the fore and aft primer information, the cutadapt software (https://github.com/marcelm/cutadapt/, accessed on 30 September 2022) was used to remove primers. Then, the paired-end clean reads were obtained. The usearch -fastq_mergepairs (V10, http://www.drive5.com/usearch/, accessed on 30 September 2022) was used to filter out tags that do not conform to default parameter and obtain raw tags. The fastp (an ultra-fast all-in-one FASTQ preprocessor, version 0.14.1, https://github.com/OpenGene/fastp, accessed on 30 September 2022) was used to clip the raw tags by sliding window quality (-W 4-M 20), and then clean tags were obtained. The Unite (for ITS, http://unite.ut.ee/index.php, accessed on 30 September 2022) database was used to annotate taxonomic information by usearch-sintax (the default confidence threshold was ≥0.8) for each representative sequence. Subsamples of these soils were used to observe electrical conductivity (EC), pH, soil organic carbon (SOC), total nitrogen (TN), total phosphorus (TP), total potassium (TK), ammonium nitrogen (NH_4_^+^-N), nitrate nitrogen (NO_3_^-^-N), available phosphorus (AP), and available potassium (AK). The ratio of SOC to TN (C:N), ratio of SOC to TP (C:P), ratio of SOC to TK (C:K), ratio of TN to TP (N:P), ratio of TN to TK (N:K), ratio of TP to TK (P:K), ratio of available nitrogen (sum of NH_4_^+^-N and NO_3_^-^-N) to AP (available N:P), ratio of available nitrogen to AK (available N:K), and ratio of AP to AK (available P:K) were determined.

### 2.3. Statistical Analyses

Correlation analyses among all the variables, including three geographic variables, four climatic variables, three plant variables (plant height, stem diameter and leaf area), and 19 soil variables (EC, pH, SOC, TN, TP, TK, NH_4_^+^-N, NO_3_^−^-N, AP, AK, C:N, C:P, C:K, N:P, N:K, P:K, available N:P, available N:K, and available P:K), were conducted, respectively. The Trophic Mode and Guild were acquired using microeco package [13,16,17]. The original FunGuild tools provide three levels of confidence ranking (i.e., ‘Highly Probable’, ‘Probable’ and ‘Possible’) data [12,13]. Referring to earlier research [12,13], only the ‘Highly Probable’ and ‘Probable’ confidence rankings function data were utilized in this study. The species and function α-diversity and β-diversity (βBray_s_: species β-diversity, βBray_f_: function β-diversity) were determined by the microeco package [13,16,17]. Species α-diversity included species richness, ACE_s_, Chao1_s_, Shannon_s_ and Simpson_s_. Function α-diversity included guild number, ACE_f_, Chao1_f_, Shannon_f_ and Simpson_f_. Phylogenetic α-diversity indices involved Faith’s phylogenetic diversity (PD) and mean nearest taxon distance (MNTD), which were determined by picante package [18]. The phylogenetic β-diversity (βMNTD) was determined by bmntd.big function of iCAMP package. Linear discriminant analysis (LDA) was utilized to determine which of taxa and ecological functions were significant among sampling sites for topsoil and subtopsoil, respectively [15]. The relative impact of environmental variable to total abundance, species, phylogenetic and function α-diversity were determined by randomForest and rfPermute packages [16]. We used mantel.partial function of vegan package to determine the partial matrix correlation between β-diversity matrix and environmental variables matrix. We used varpart function to split the variations of total abundance, the α- and β-diversity into four elucidatory parts (i.e., geographic variables, climatic variables, soil variables, and plant variables) [2,10,19,20,21,22].

## 3. Results

### 3.1. Spatial Distributions of Environmental Variables, and Total Abundance and Diversity of Soil Fungal Community

**Figure A2:**
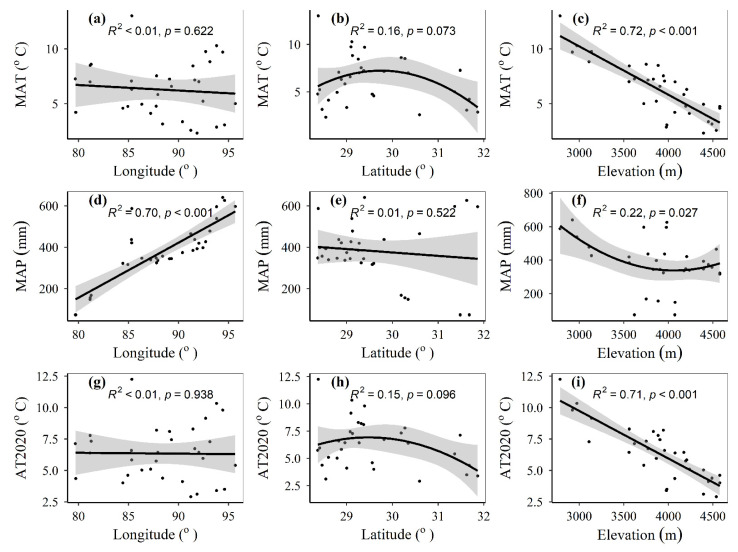

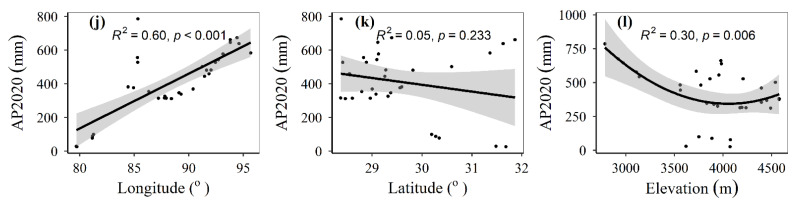
Relationships between (**a**) mean annual temperature (MAT) and longitude, (**b**) MAT and latitude, (**c**) MAT and elevation, (**d**) mean annual precipitation (MAP) and longitude, (**e**) MAP and latitude, (**f**) MAP and elevation, (**g**) annual temperature in 2020 (AT2020) and longitude, (**h**) AT2020 and latitude, (**i**) AT2020 and elevation, (**j**) annual precipitation in 2020 (AP2020) and longitude, (**k**) AP2020 and latitude, and (**l**) AP2020 and elevation.

**Figure A3:**
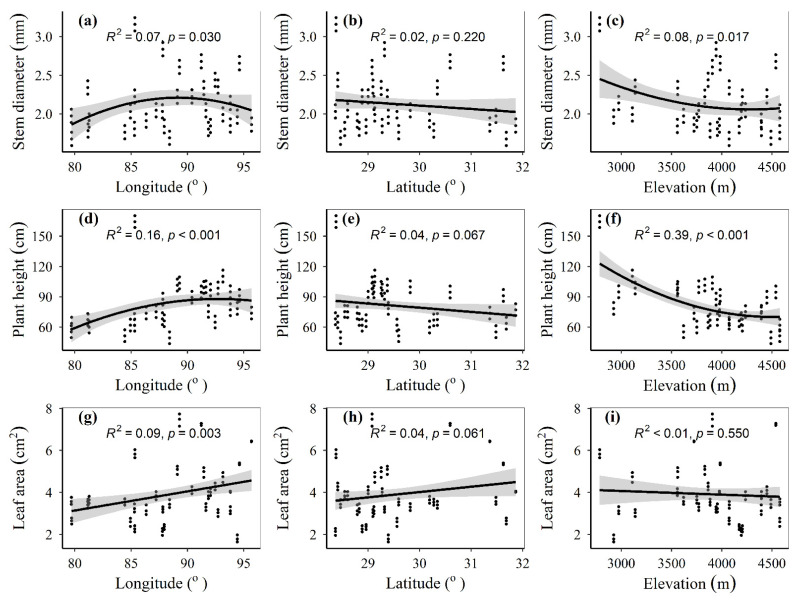
Relationships between (**a**) stem diameter and longitude, (**b**) stem diameter and latitude, (**c**) stem diameter and elevation, (**d**) plant height and longitude, (**e**) plant height and latitude, (**f**) plant height and elevation, (**g**) leaf area and longitude, (**h**) leaf area and latitude, and (**i**) leaf area and elevation.

**Figure A4:**
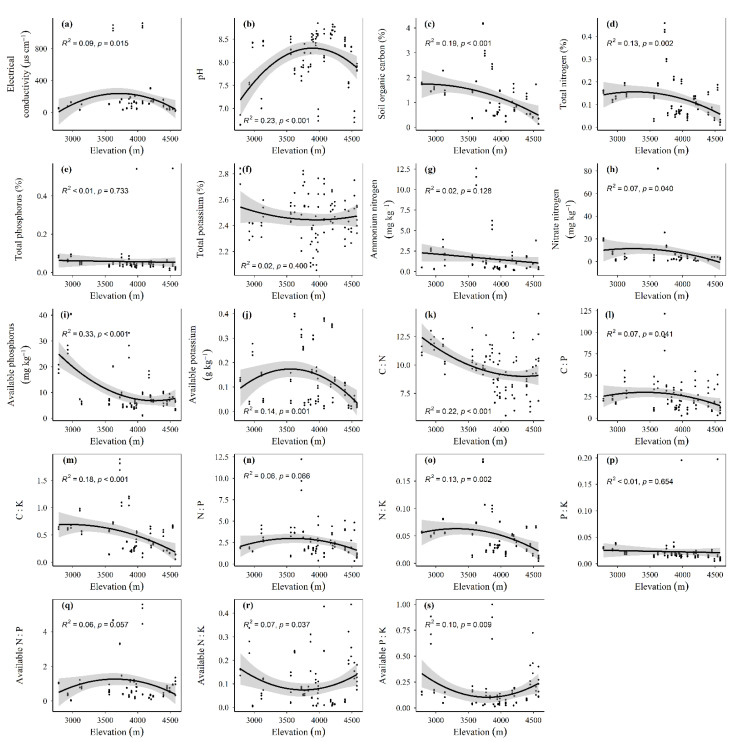
Relationships of elevation with soil (**a**) electrical conductivity, (**b**) pH, (**c**) soil organic carbon (SOC), (**d**) total nitrogen (TN), (**e**) total phosphorus (TP), (**f**) total potassium (TK), (**g**) ammonium nitrogen (NH_4_^+^-N), (**h**) nitrate nitrogen (NO_3_^−^-N), (**i**) available phosphorus (AP), (**j**) available potassium (AK), (**k**) ratio of SOC to TN (C:N), (**l**) ratio of SOC to TP (C:P), (**m**) ratio of SOC to TK (C:K), (**n**) ratio of TN to TP (N:P), (**o**) ratio of TN to TK (N:K), (**p**) ratio of TP to TK (P:K), (**q**) ratio of available nitrogen to AP (available N:P), (**r**) ratio of available nitrogen to AK (available N:K) and (**s**) ratio of AP to AK (available P:K) at a depth of 0–10 cm.

**Figure A5:**
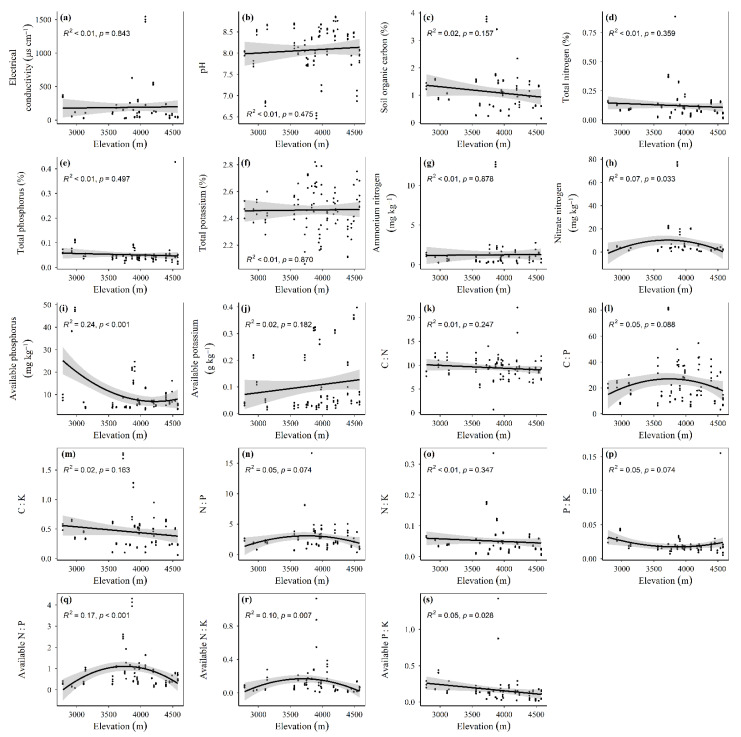
Relationships of elevation with soil (**a**) electrical conductivity, (**b**) pH, (**c**) soil organic carbon (SOC), (**d**) total nitrogen (TN), (**e**) total phosphorus (TP), (**f**) total potassium (TK), (**g**) ammonium nitrogen (NH_4_^+^-N), (**h**) nitrate nitrogen (NO_3_^−^-N), (**i**) available phosphorus (AP), (**j**) available potassium (AK), (**k**) ratio of SOC to TN (C:N), (**l**) ratio of SOC to TP (C:P), (**m**) ratio of SOC to TK (C:K), (**n**) ratio of TN to TP (N:P), (**o**) ratio of TN to TK (N:K), (**p**) ratio of TP to TK (P:K), (**q**) ratio of available nitrogen to AP (available N:P), (**r**) ratio of available nitrogen to AK (available N:K) and (**s**) ratio of AP to AK (available P:K) at a depth of 10–20 cm.

**Figure A6:**
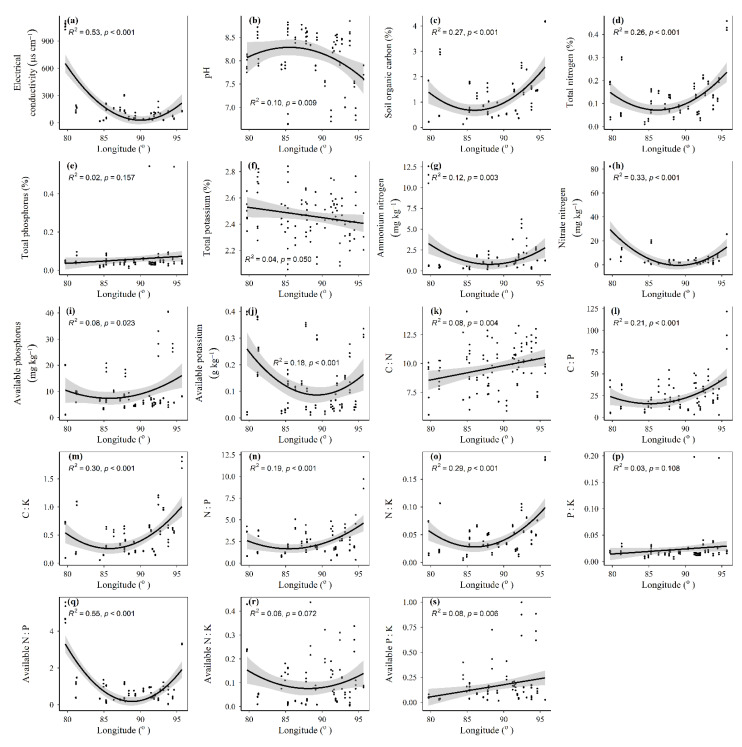
Relationships of longitude with soil (**a**) electrical conductivity, (**b**) pH, (**c**) soil organic carbon (SOC), (**d**) total nitrogen (TN), (**e**) total phosphorus (TP), (**f**) total potassium (TK), (**g**) ammonium nitrogen (NH_4_^+^-N), (**h**) nitrate nitrogen (NO_3_^−^-N), (**i**) available phosphorus (AP), (**j**) available potassium (AK), (**k**) ratio of SOC to TN (C:N), (**l**) ratio of SOC to TP (C:P), (**m**) ratio of SOC to TK (C:K), (**n**) ratio of TN to TP (N:P), (**o**) ratio of TN to TK (N:K), (**p**) ratio of TP to TK (P:K), (**q**) ratio of available nitrogen to AP (available N:P), (**r**) ratio of available nitrogen to AK (available N:K) and (**s**) ratio of AP to AK (available P:K) at a depth of 0–10 cm.

**Figure A7:**
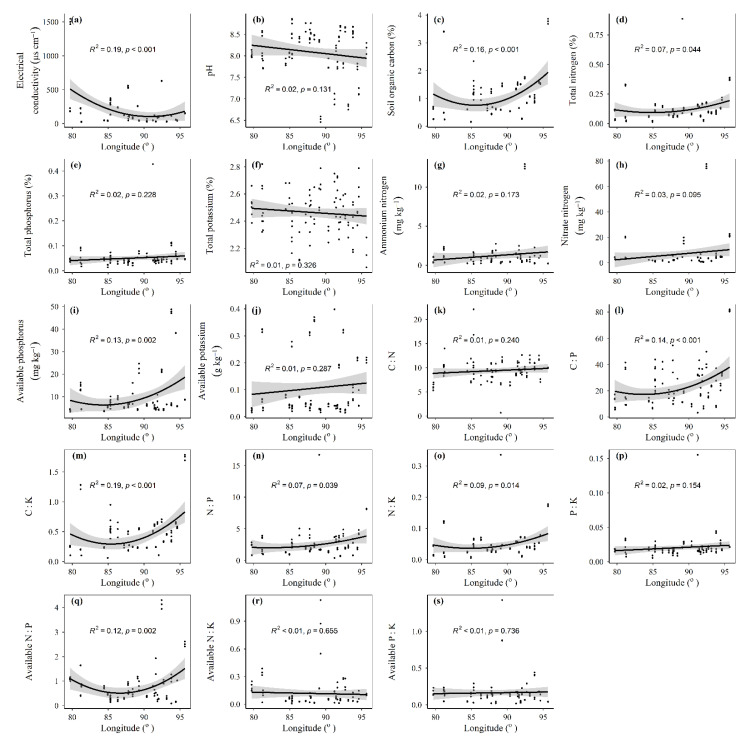
Relationships of longitude with soil (**a**) electrical conductivity, (**b**) pH, (**c**) soil organic carbon (SOC), (**d**) total nitrogen (TN), (**e**) total phosphorus (TP), (**f**) total potassium (TK), (**g**) ammonium nitrogen (NH_4_^+^-N), (**h**) nitrate nitrogen (NO_3_^−^-N), (**i**) available phosphorus (AP), (**j**) available potassium (AK), (**k**) ratio of SOC to TN (C:N), (**l**) ratio of SOC to TP (C:P), (**m**) ratio of SOC to TK (C:K), (**n**) ratio of TN to TP (N:P), (**o**) ratio of TN to TK (N:K), (**p**) ratio of TP to TK (P:K), (**q**) ratio of available nitrogen to AP (available N:P), (**r**) ratio of available nitrogen to AK (available N:K) and (**s**) ratio of AP to AK (available P:K) at a depth of 10–20 cm.

**Figure A8:**
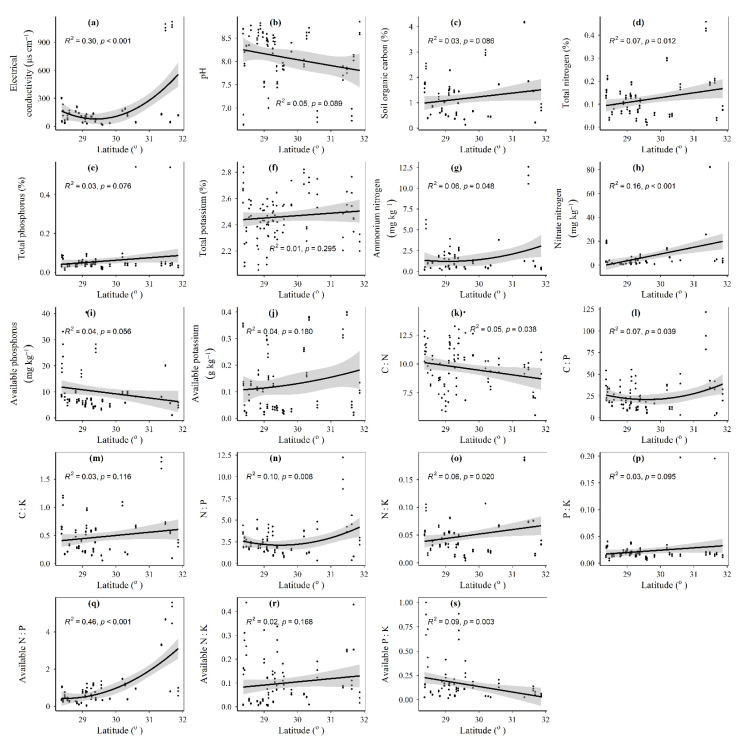
Relationships of latitude with soil (**a**) electrical conductivity, (**b**) pH, (**c**) soil organic carbon (SOC), (**d**) total nitrogen (TN), (**e**) total phosphorus (TP), (**f**) total potassium (TK), (**g**) ammonium nitrogen (NH_4_^+^-N), (**h**) nitrate nitrogen (NO_3_^−^-N), (**i**) available phosphorus (AP), (**j**) available potassium (AK), (**k**) ratio of SOC to TN (C:N), (**l**) ratio of SOC to TP (C:P), (**m**) ratio of SOC to TK (C:K), (**n**) ratio of TN to TP (N:P), (**o**) ratio of TN to TK (N:K), (**p**) ratio of TP to TK (P:K), (**q**) ratio of available nitrogen to AP (available N:P), (**r**) ratio of available nitrogen to AK (available N:K) and (**s**) ratio of AP to AK (available P:K) at a depth of 0–10 cm.

**Figure A9:**
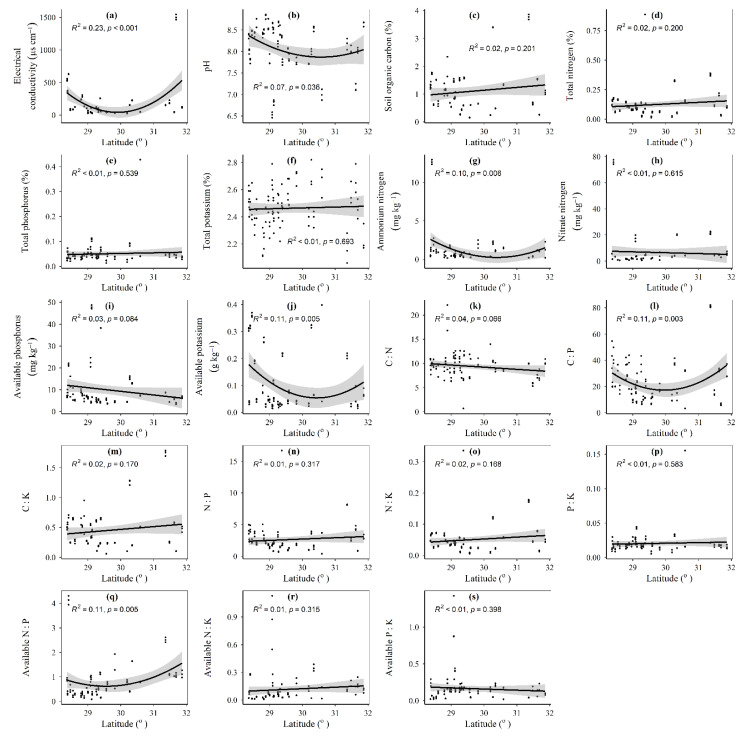
Relationships of latitude with soil (**a**) electrical conductivity, (**b**) pH, (**c**) soil organic carbon (SOC), (**d**) total nitrogen (TN), (**e**) total phosphorus (TP), (**f**) total potassium (TK), (**g**) ammonium nitrogen (NH_4_^+^-N), (**h**) nitrate nitrogen (NO_3_^−^-N), (**i**) available phosphorus (AP), (**j**) available potassium (AK), (**k**) ratio of SOC to TN (C:N), (**l**) ratio of SOC to TP (C:P), (**m**) ratio of SOC to TK (C:K), (**n**) ratio of TN to TP (N:P), (**o**) ratio of TN to TK (N:K), (**p**) ratio of TP to TK (P:K), (**q**) ratio of available nitrogen to AP (available N:P), (**r**) ratio of available nitrogen to AK (available N:K) and (**s**) ratio of AP to AK (available P:K) at a depth of 10–20 cm.

The MAT and AT2020 exhibited quadratic tendencies with latitude from 28.37° N to 31.86° N but decreased linearly with elevation from 2785 m to 4578 m (Figure A2). The MAP and AP2020 increased linearly with longitude from 79.69° E to 95.68° E, but exhibited quadratic correlations with elevation from 2785 m to 4578 m (Figure A2). Both stem diameter and plant height exhibited quadratic correlations with longitude (79.69°–95.68° E) and elevation (2785–4578 m) (Figure A3). Leaf area increased with longitude from 79.69° E to 95.68° E (Figure A3). The correlations between the 19 soil variables and the 3 geographic variables are presented in Figure A4, Figure A5, Figure A6, Figure A7, Figure A8 and Figure A9.

**Figure A10:**
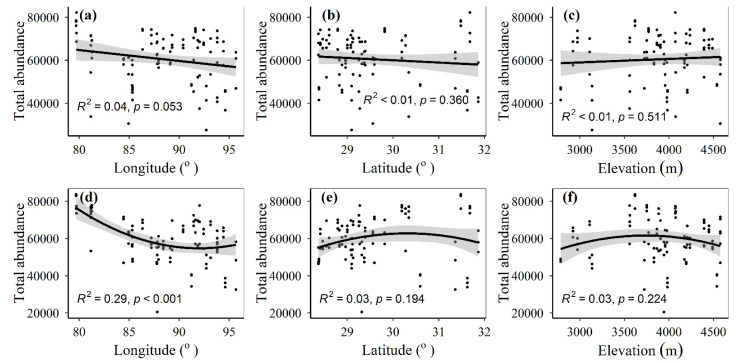
Relationships between total abundance and geographic variables (longitude, latitude, and elevation) at (**a**–**c**) 0–10 cm and (**d**–**f**) 10–20 cm depth.

**Figure A11:**
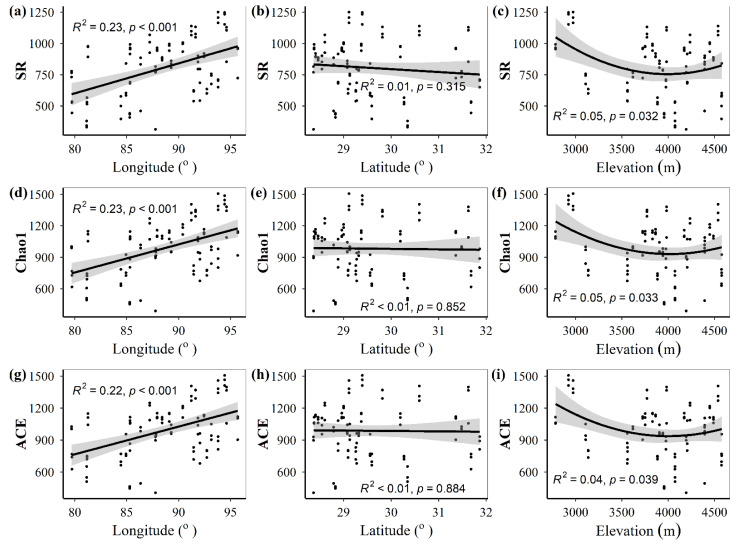

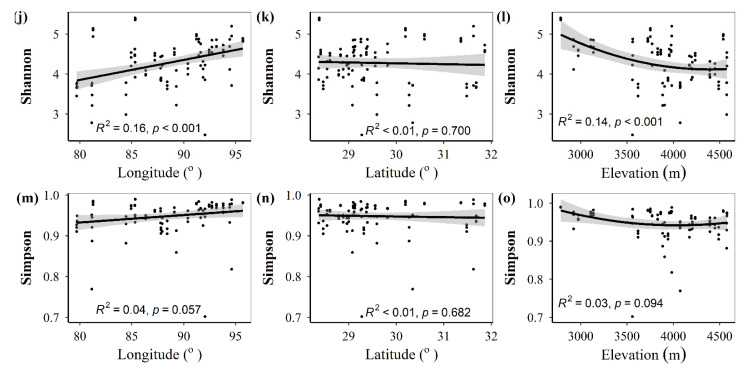
Relationships between species α-diversity and geographic variables (longitude, latitude, and elevation) at 0–10 cm depth. (**a**–**c**) species richness (SR), (**d**–**f**) Chao1, (**g**–**i**) ACE, (**j**–**l**) Shannon, and (**m**–**o**) Simpson.

**Figure A12:**
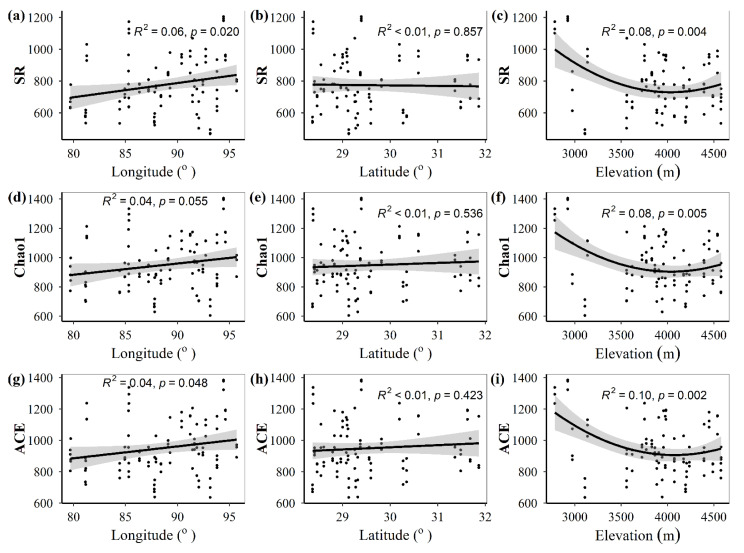

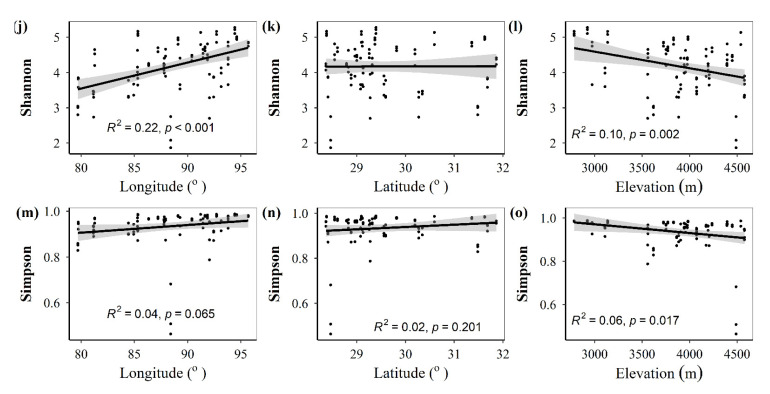
Relationships between species α-diversity and geographic variables (longitude, latitude, and elevation) at 10–20 cm depth. (**a**–**c**) species richness (SR), (**d**–**f**) Chao1, (**g**–**i**) ACE, (**j**–**l**) Shannon, and (**m**–**o**) Simpson.

**Figure A13:**
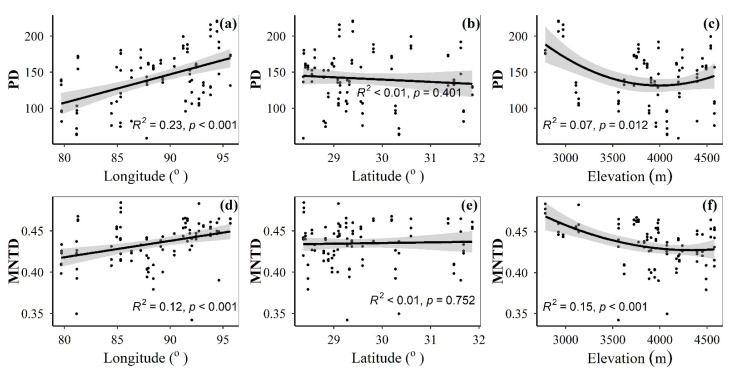
Relationships between phylogenetic α-diversity and geographic variables (longitude, latitude, and elevation) at 0–10 cm depth. (**a**–**c**) phylogenetic diversity (PD), and (**d**–**f**) mean nearest taxon distance (MNTD).

**Figure A14:**
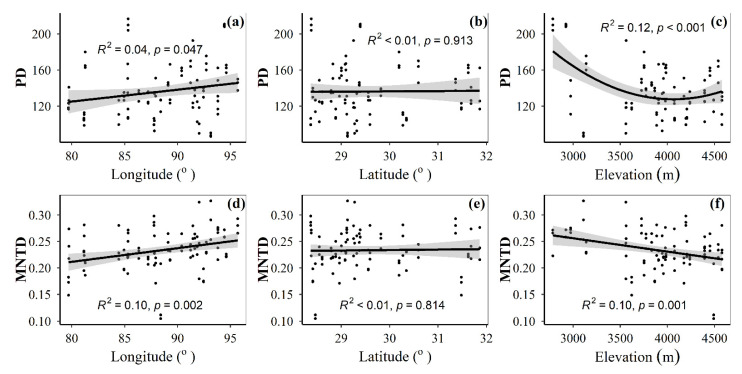
Relationships between phylogenetic α-diversity and geographic variables (longitude, latitude, and elevation) at 10–20 cm depth. (**a**–**c**) phylogenetic diversity (PD), and (**d**–**f**) mean nearest taxon distance (MNTD).

**Figure A15:**
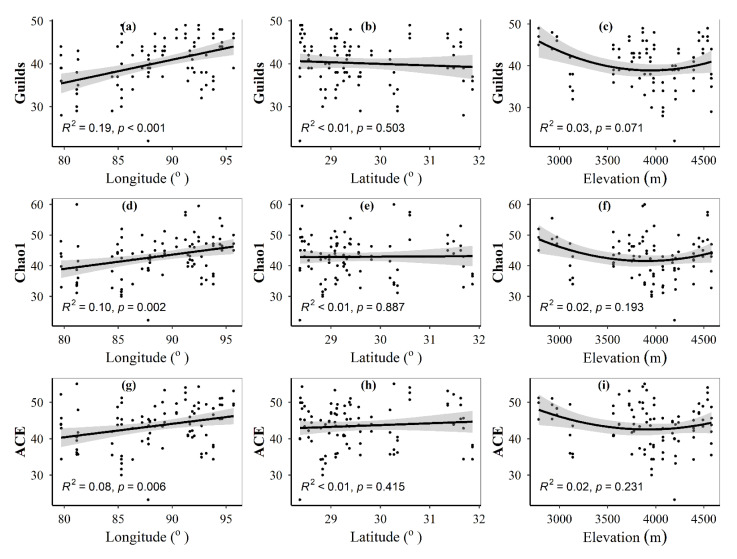

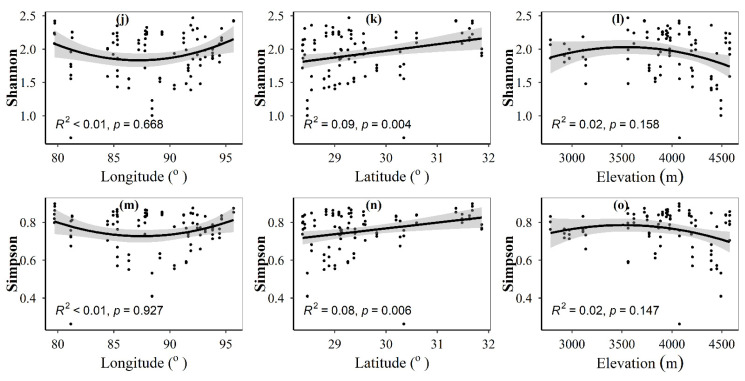
Relationships between functional α-diversity and geographic variables (longitude, latitude, and elevation) at 0–10 cm depth. (**a**–**c**) numbers of FunGuild at guild level (Guilds), (**d**–**f**) Chao1, (**g**–**i**) ACE, (**j**–**l**) Shannon, and (**m**–**o**) Simpson.

**Figure A16:**
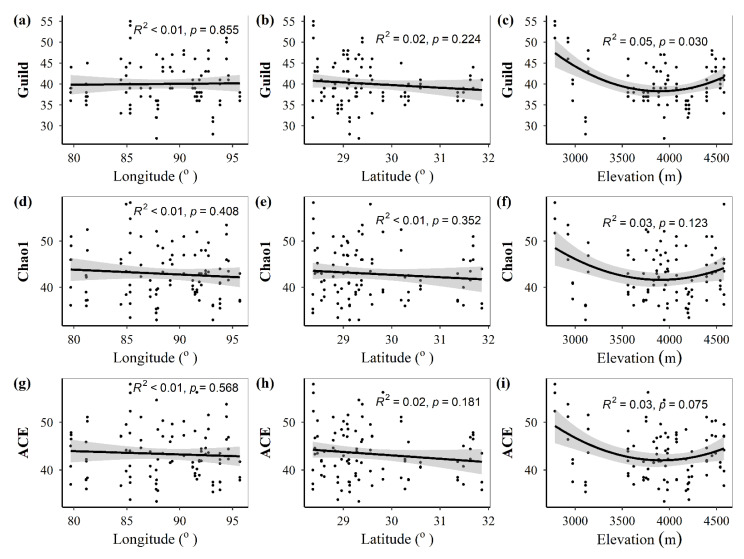

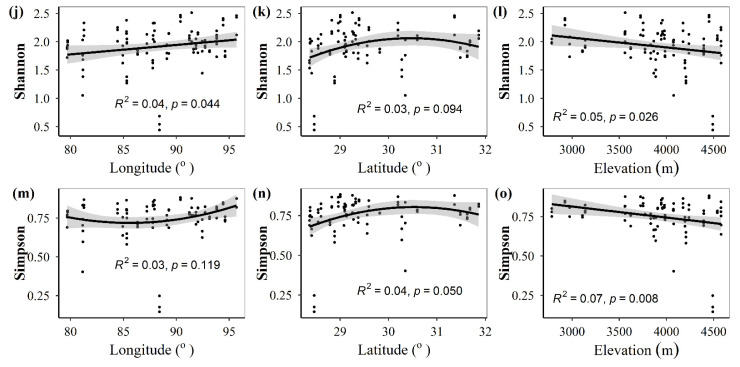
Relationships between functional α-diversity and geographic variables (longitude, latitude, and elevation) at 10–20 cm depth. (**a**–**c**) numbers of FunGuild at guild level (Guilds), (**d**–**f**) Chao1, (**g**–**i**) ACE, (**j**–**l**) Shannon, and (**m**–**o**) Simpson.

Different α-diversity indices had different relationships with longitude, latitude, and elevation (Figure A10, Figure A11, Figure A12, Figure A13, Figure A14, Figure A15 and Figure A16). Species, phylogenetic and function β-diversity had marginally significant or significant correlations with longitude (Table 1). Species β-diversity increased with increasing latitude and elevation (Table 1).

### 3.2. Correlations between Fungal Community and Environmental Factors

**Figure 1:**
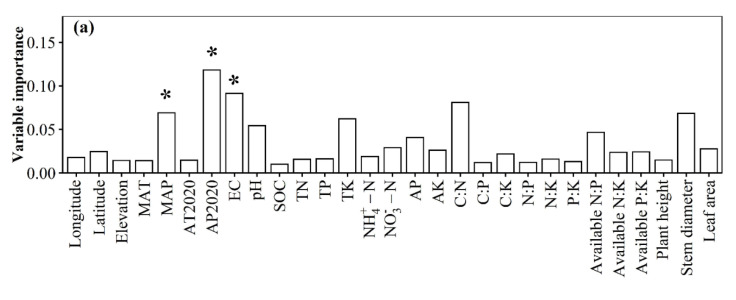

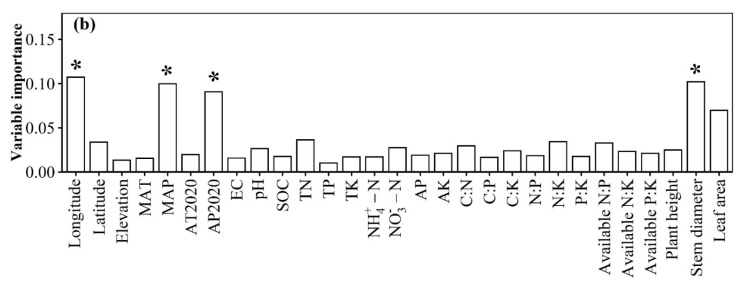
Relative impacts of environmental variables on total abundance of soil fungal community for (**a**) topsoil and (**b**) subtopsoil. The * above the bar indicated this variable had a significant influence on total abundance of soil fungal community at *p* < 0.05 level. MAT: mean annual temperature in 1982–2020; MAP: mean annual precipitation in 1982–2020; AT2020: annual temperature in 2020; AP2020: annual precipitation in 2020; EC: soil electrical conductivity; pH: soil pH; SOC: soil organic carbon; TN: soil total nitrogen; TP: soil total phosphorus; TK: soil total potassium; NH_4_^+^-N: soil ammonium nitrogen; NO_3_^−^-N: soil nitrate nitrogen; AP: soil available phosphorus; AK: soil available potassium; C:N: ratio of SOC to TN; C:P: ratio of SOC to TP; C:K: ratio of SOC to TK; N:P: ratio of TN to TP; N:K: ratio of TN to TK; P:K: ratio of TP to TK; available N:P: ratio of available nitrogen to phosphorus; available N:K: ratio of available nitrogen to potassium; available P:K: ratio of available phosphorus to potassium.

**Figure 2:**
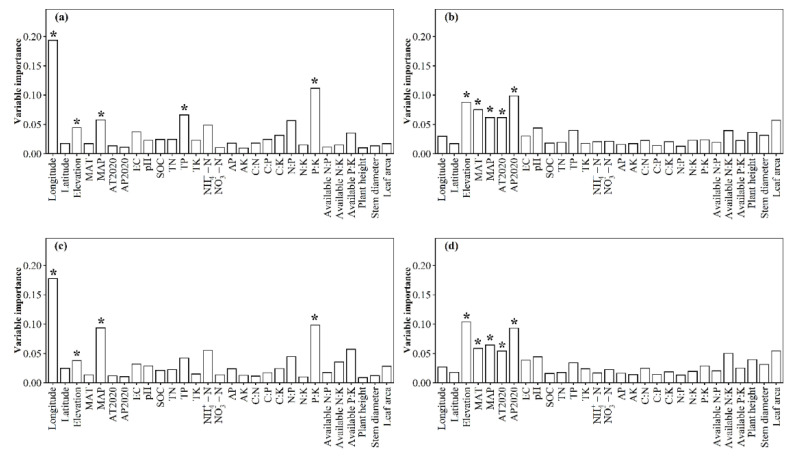

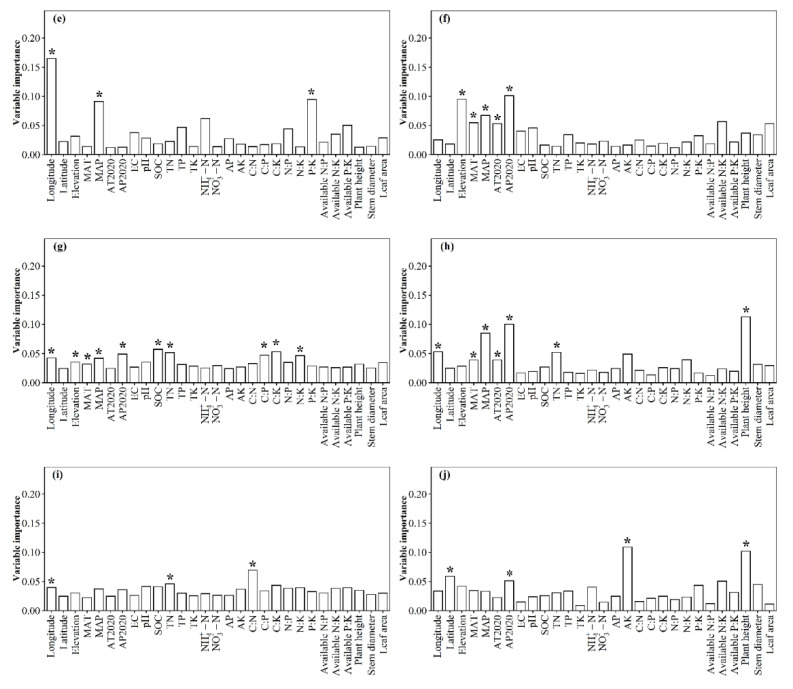
Relative impacts of environmental variables on (**a**,**b**) operational taxonomic units (OTU_s_), (**c**,**d**) Chao1_s_, (**e**,**f**) ACE_s_, (**g**,**h**) Shannon_s_ and (**i**,**j**) Simpson_s_ for (**a**,**c**,**e**,**g**,**i**) topsoil and (**b**,**d**,**f**,**h**,**j**) subtopsoil, respectively. The * above the bar indicated this variable had a significant influence on species α-diversity of soil fungal community at *p* < 0.05 level. MAT: mean annual temperature in 1982–2020; MAP: mean annual precipitation in 1982–2020; AT2020: annual temperature in 2020; AP2020: annual precipitation in 2020; EC: soil electrical conductivity; pH: soil pH; SOC: soil organic carbon; TN: soil total nitrogen; TP: soil total phosphorus; TK: soil total potassium; NH_4_^+^-N: soil ammonium nitrogen; NO_3_^−^-N: soil nitrate nitrogen; AP: soil available phosphorus; AK: soil available potassium; C:N: ratio of SOC to TN; C:P: ratio of SOC to TP; C:K: ratio of SOC to TK; N:P: ratio of TN to TP; N:K: ratio of TN to TK; P:K: ratio of TP to TK; available N:P: ratio of available nitrogen to phosphorus; available N:K: ratio of available nitrogen to potassium; available P:K: ratio of available phosphorus to potassium.

**Figure 3:**
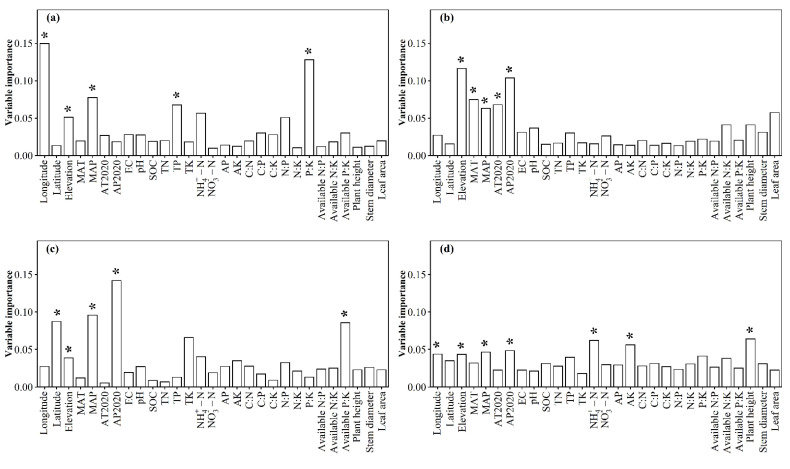
Relative impacts of environmental variables on (**a**,**b**) Faith’s phylogenetic diversity (PD), and (**c**,**d**) mean nearest taxon distance (MNTD) for (**a**,**c**) topsoil and (**b**,**d**) subtopsoil, respectively. The * above the bar indicated this variable had a significant influence on phylogenetic α-diversity of soil fungal community at *p* < 0.05 level. MAT: mean annual temperature in 1982–2020; MAP: mean annual precipitation in 1982–2020; AT2020: annual temperature in 2020; AP2020: annual precipitation in 2020; EC: soil electrical conductivity; pH: soil pH; SOC: soil organic carbon; TN: soil total nitrogen; TP: soil total phosphorus; TK: soil total potassium; NH_4_^+^-N: soil ammonium nitrogen; NO_3_^−^-N: soil nitrate nitrogen; AP: soil available phosphorus; AK: soil available potassium; C:N: ratio of SOC to TN; C:P: ratio of SOC to TP; C:K: ratio of SOC to TK; N:P: ratio of TN to TP; N:K: ratio of TN to TK; P:K: ratio of TP to TK; available N:P: ratio of available nitrogen to phosphorus; available N:K: ratio of available nitrogen to potassium; available P:K: ratio of available phosphorus to potassium.

**Figure 4:**
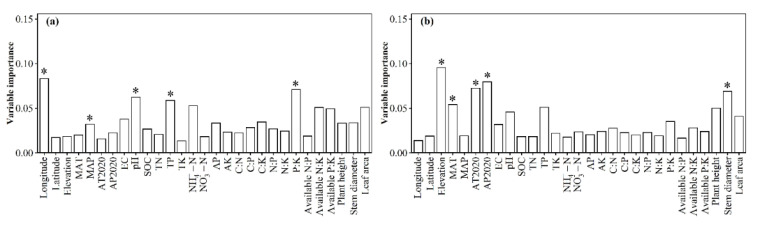

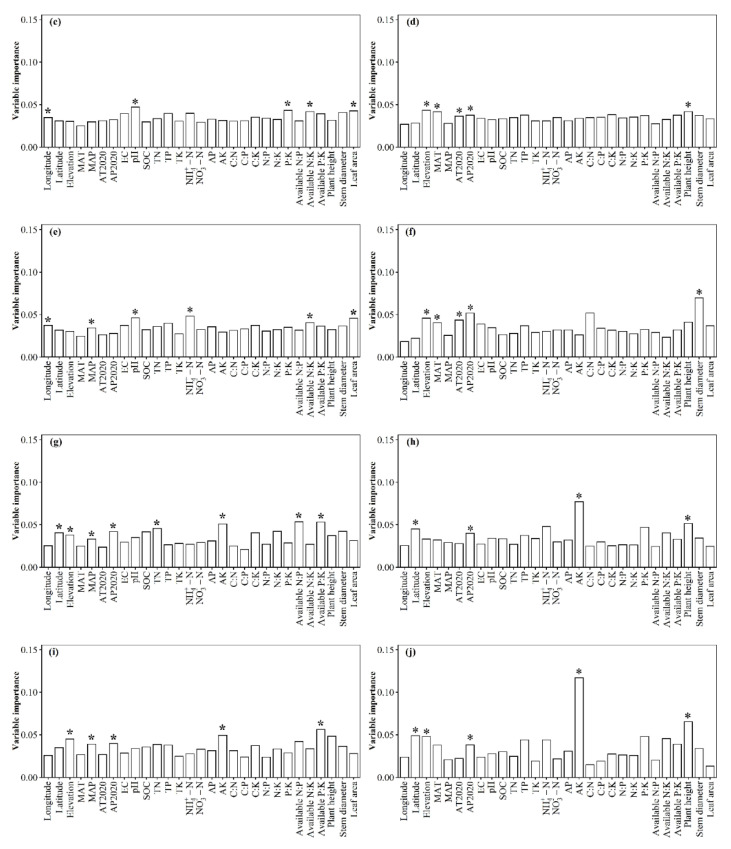
Relative impacts of environmental variables on (**a**,**b**) guild numbers, (**c**,**d**) Chao1_f_, (**e**,**f**) ACE_f_, (**g**,**h**) Shannon_f_ and (**i**,**j**) Simpson_f_ for (**a**,**c**,**e**,**g**,**i**) topsoil and (**b**,**d**,**f**,**h**,**j**) subtopsoil, respectively. The * above the bar indicated this variable had a significant influence on function α-diversity of soil fungal community at *p* < 0.05 level. MAT: mean annual temperature in 1982–2020; MAP: mean annual precipitation in 1982–2020; AT2020: annual temperature in 2020; AP2020: annual precipitation in 2020; EC: soil electrical conductivity; pH: soil pH; SOC: soil organic carbon; TN: soil total nitrogen; TP: soil total phosphorus; TK: soil total potassium; NH_4_^+^-N: soil ammonium nitrogen; NO_3_^−^-N: soil nitrate nitrogen; AP: soil available phosphorus; AK: soil available potassium; C:N: ratio of SOC to TN; C:P: ratio of SOC to TP; C:K: ratio of SOC to TK; N:P: ratio of TN to TP; N:K: ratio of TN to TK; P:K: ratio of TP to TK; available N:P: ratio of available nitrogen to phosphorus; available N:K: ratio of available nitrogen to potassium; available P:K: ratio of available phosphorus to potassium.

**Figure 5:**
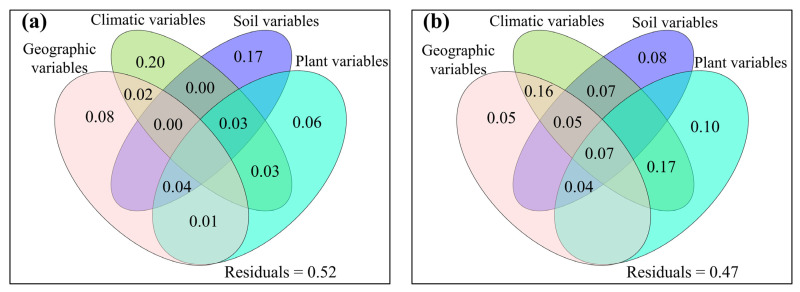
The mutual as well as excluded impacts of geographic variables, climatic variables, soil variables and plant variables on total abundance for (**a**) topsoil and (**b**) subtopsoil, respectively.

**Figure 6:**
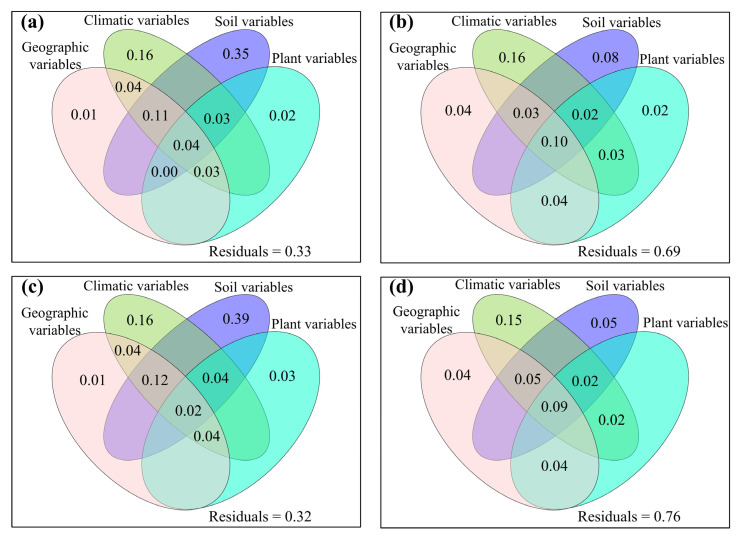

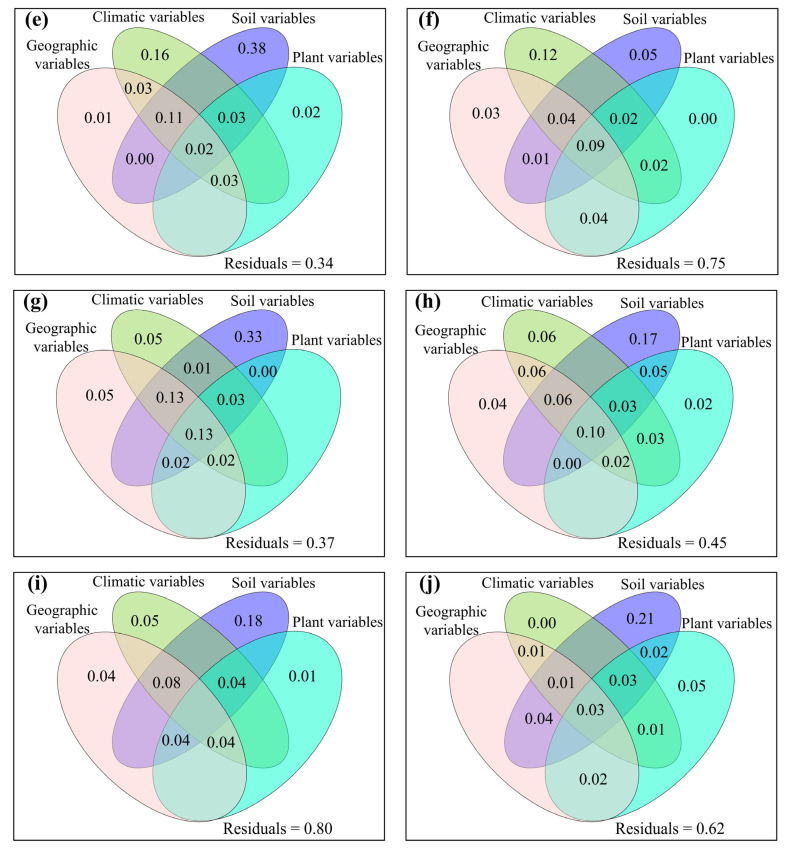
The mutual as well as excluded impacts of geographic variables, climatic variables, soil variables and plant variables on (**a**,**b**) operational taxonomic units (OTU_s_), (**c**,**d**) Chao1_s_, (**e**,**f**) ACE_s_, (**g**,**h**) Shannon_s_ and (**i**,**j**) Simpson_s_ of soil fungal community for (**a**,**c**,**e**,**g**,**i**) topsoil and (**b**,**d**,**f**,**h**,**j**) subtopsoil, respectively.

**Figure 7:**
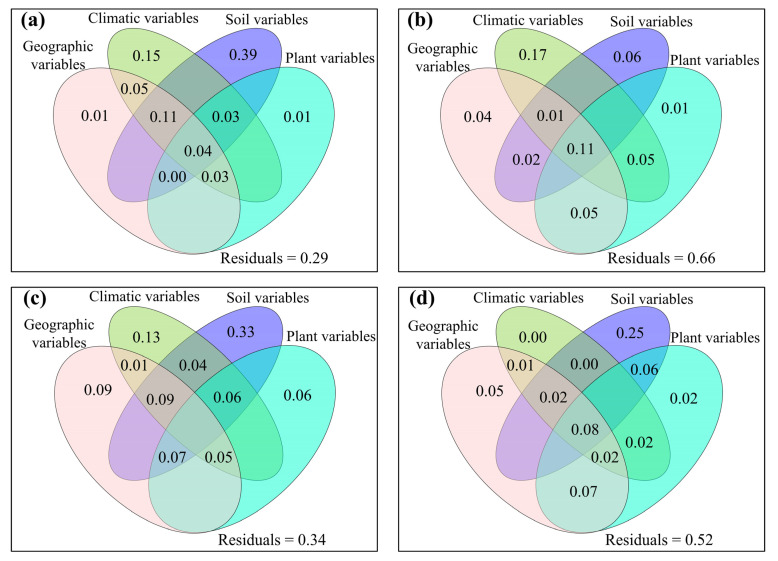
The mutual as well as excluded impacts of geographic variables, climatic variables, soil variables and plant variables on (**a**,**b**) Faith’s phylogenetic diversity (PD), and (**c**,**d**) mean nearest taxon distance (MNTD) of soil fungal community for (**a**,**c**) topsoil and (**b**,**d**) subtopsoil, respectively.

**Figure 8:**
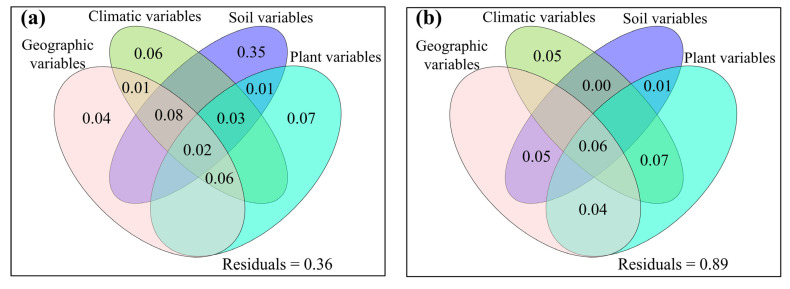

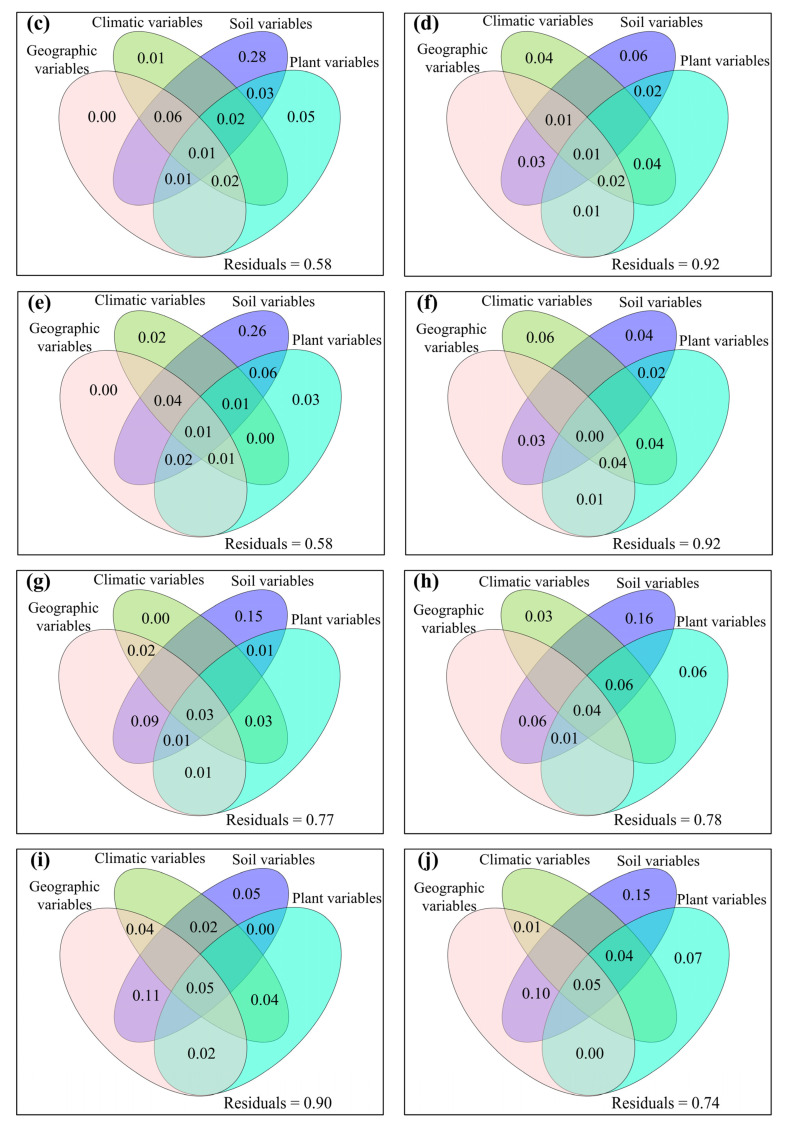
The mutual as well as excluded impacts of geographic variables, climatic variables, soil variables and plant variables on (**a**,**b**) guild numbers, (**c**,**d**) Chao1_f_, (**e**,**f**) ACE_f_, (**g**,**h**) Shannon_f_ and (**i**,**j**) Simpson_f_ of soil fungal community for (**a**,**c**,**e**,**g**,**i**) topsoil and (**b**,**d**,**f**,**h**,**j**) subtopsoil, respectively.

**Figure 9:**
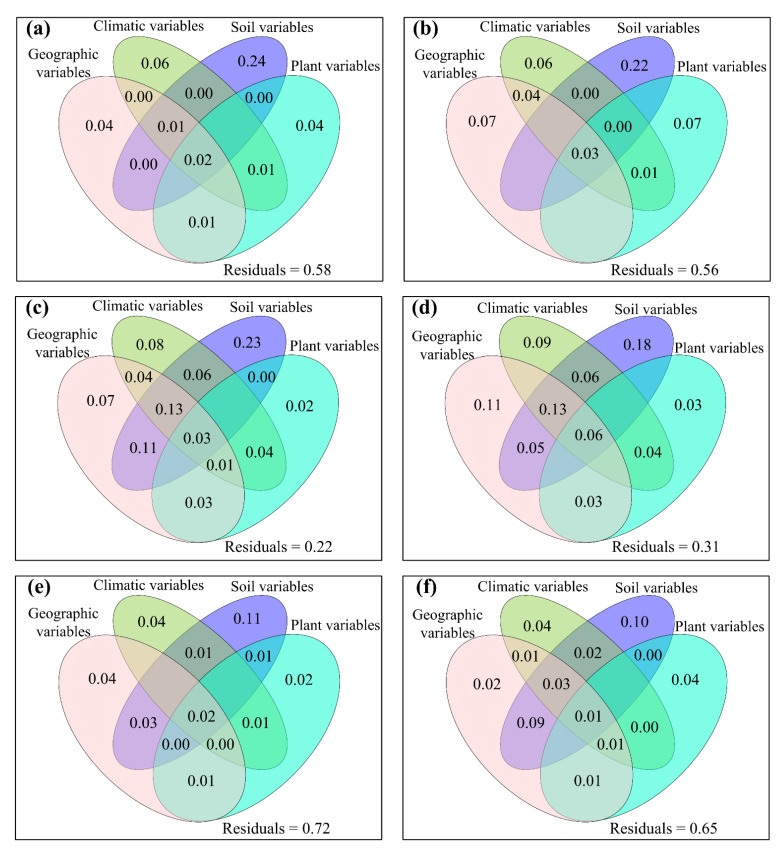
The mutual as well as excluded impacts of geographic variables, climatic variables, soil variables and plant variables on (**a**,**b**) species β-diversity, (**c**,**d**) phylogenetic β-diversity, and (**e**,**f**) function β-diversity of soil fungal community for (**a**,**c**,**e**) topsoil and (**b**,**d**,**f**) subtopsoil, respectively.

The relative impacts of environmental variables to total abundance andα-diversity varied (Figure 1, Figure 2, Figure 3 and Figure 4). Different α-diversity indices had different predominated factors (Figure 2 and Figure 3). Species, phylogenetic and function β-diversity had dissimilar correlations with environmental variable, and their predominated factors also differed (Table 1). The relative impacts of geographic, climatic, variables and variables to total abundance and diversity were elucidated in Figure 5, Figure 6, Figure 7, Figure 8 and Figure 9. Climatic variables and plant variables had the greatest and least excluded impacts on total abundance for topsoil, respectively (Figure 5). In contrast, climatic variables and plant variables had the least and greatest excluded impacts on total abundance for subtopsoil, respectively (Figure 5). Soil variables had the greatest excluded impacts on species, phylogenetic and function α- and β-diversity for topsoil (Figure 6, Figure 7, Figure 8 and Figure 9). Climatic variables had the greatest excluded impacts on OTU_s_, Chao1_s_, ACE_s_, PD, Guild numbers and ACE_f_, but soil variables had the greatest excluded impacts on Shannon_s_, Simpson_s_, Chao1_f_, MNTD, Shannon_f_, Simpson_f_, species, phylogenetic and function β-diversity for subtopsoil (Figure 6, Figure 7, Figure 8 and Figure 9).

## 4. Discussion

Based on our discoveries, the total abundance and diversity of soil fungal community overall had less correlations with latitude than longitude and elevation. This discovery could be explained by at least one of the following reasons. Firstly, the elevation range, longitude span, and latitude span were 1793 m (2785–4578 m), 15.99° (79.69–95.68°), and 3.49° (28.37–31.86°), respectively. Secondly, temperature and/or precipitation, which can modify the total abundance and diversity of soil fungal community [13,23], had lower correlations with latitude than longitude and elevation. Thirdly, plant height, stem diameter and/or leaf area, which might also modify total abundance and diversity of soil fungal community (Figure 1, Figure 2, Figure 3, Figure 4, Figure 5, Figure 6, Figure 7, Figure 8 and Figure 9), had less correlations with latitude than longitude and elevation (Figure A3).

Based on our discoveries, the geo-distribution patterns of total abundance and diversity of soil fungal community for topsoil were dissimilar from those for subtopsoil. Similarly, some earlier studies found that soil fungal community at different soil depths had different responses to external disturbance on the Tibetan Plateau [13,16,24]. All these cautioned that investigating soil microbial community in single soil depth may not fully reflect the spatial distribution pattern of soil microbial community structure and function, and the response mechanism of soil microbial community to external disturbance. It is necessary to strengthen the study of soil microbial community at multiple soil depths. This finding could be explained by at least one of the following reasons. Firstly, both soil temperature as well as moisture can alter soil fungal abundance, α-diversity, and community composition [13,16,25]. Both soil temperature as well as moisture can adjust with soil depth [13,24]. On the other hand, climate change (e.g., global warming; precipitation change) can alter soil temperature as well as soil moisture, and their change magnitudes can adjust with soil depth [13,26,27]. Secondly, soil carbon, nitrogen, phosphorus, potassium contents and obtainability, as well as their ratios might also alter soil fungal abundance, α-diversity and community composition [14,15]. The correlations between these soil variables and total abundance α-diversity and community composition of soil fungal community varied with soil depth in the current study. On the other hand, global warming as well as precipitation change can also result in the changes in soil carbon, nitrogen, phosphorus and potassium, and their change levels can also adjust with soil depth [28]. Thirdly, the total abundance as well as diversity of soil fungal community might be also correlated with soil mechanical composition, soil bulk density, and/or soil compaction [16], which might also adjust with soil depth.

Based on our discoveries, the geo-distribution patterns of soil fungal species, phylogenetic and function diversity of *P. flaccidum* were not completely the same. For example, species and function β-diversity had closer correlations with longitude than phylogenetic β-diversity (Table 1). This discovery was similar to earlier reports [12,13,14,15,16,29,30]. This discovery cautioned that a single aspect of diversity cannot completely reveal the geo-distribution patterns of soil fungal diversity and could be explained by at least one of the succeeding reasons. Firstly, species, phylogenetic and function diversity can represent biodiversity from dissimilar perspectives, and they are fundamentally dissimilar [18,31]. Dissimilar species might have similar phylogenetic information and/or biological functions [32,33,34]. Secondly, both temperature and water obtainability can modify soil fungal diversity [14,16], which was strengthened by the current study. Soil fungal species, phylogenetic and function diversity can have dissimilar correlations with temperature as well as water obtainability [13,16]. Thirdly, soil variables (e.g., pH) can also modify soil fungal diversity [14]. Soil fungal species, phylogenetic and function diversity can have dissimilar correlations with soil variables. Fourthly, soil fungal diversity can be also closely correlated with plant variables [14], which can have dissimilar correlations with soil fungal species, phylogenetic and function diversity [12,14].

Based on our discoveries, compared to temperature, precipitation had stronger impacts on total abundance, species α-diversity, phylogenetic α-diversity, and function β-diversity of soil fungal community for both topsoil and subtopsoil. Furthermore, compared to temperature, precipitation had stronger impacts on function α-diversity for topsoil, species β-diversity for topsoil, and phylogenetic β-diversity for subtopsoil. These discoveries were similar with earlier reports [2,4,27], and further supported that the Tibetan Plateau is a cold and dry region [35]. Furthermore, this phenomenon cautioned that we should not only pay attention to the impacts of global warming on the Qinghai-Tibet Plateau, but also pay attention to the impacts of precipitation change on the Tibetan Plateau. More field experiments of precipitation change are needed to better capture the impacts of future climate change on the Qinghai-Tibet Plateau.

## 5. Conclusions

The geo-distribution patterns for soil fungal community of *P. flaccidum* were explored along 32 sites in alpine areas across Tibet. Total abundance and α- and β-diversity of soil fungal community overall had lower correlations with latitude than longitude and elevation. The geo-distribution pattern of soil fungal community at 0–10 cm depth was different from that at 10–20 cm depth. Soil fungal species, phylogenetic and function diversity had dissimilar geo-distribution patterns, indicating that it is necessary to combine species, phylogenetic and function diversity to better reflect geo-distribution patterns of soil fungal community. This study warned that both global warming and precipitation change can affect soil fungal communities of *P. flaccidum*, but the effect of precipitation change should be given more attention.

## Figures and Tables

**Table 1:** Partial mantel tests between β-diversity and environmental variables.

Soil Depth	Variables	Species β- Diversity	Phylogenetic β- Diversity	Function β- Diversity
0–10 cm	Longitude	0.19 ***	0.06 ^+^	0.13 **
	Latitude	0.16 ***	−0.01	0.03
	Elevation	0.24 ***	0.01	0.04
	MAT	0.07 *	0.08 *	0.03
	MAP	0.16 ***	0.00	0.12 *
	AT2020	0.04	0.03	0.03
	AP2020	0.16 ***	0.08 ^+^	0.12 *
	EC	0.22***	−0.07	0.15 **
	pH	0.19 ***	0.13**	−0.08
	SOC	0.19 ***	−0.07	0.16 ***
	TN	0.20 ***	−0.10	0.20 ***
	TP	−0.05	−0.07	0.04
	TK	0.13 ***	0.10 *	−0.05
	NH_4_^+^-N	0.05 ^+^	0.08 *	0.01
	NO_3_^−^-N	0.04	0.08 ^+^	−0.02
	AP	−0.05	−0.03	−0.01
	AK	0.12 ***	−0.05	0.09 **
	C:N	0.04	−0.04	0.06 ^+^
	C:P	0.16 ***	−0.07	0.06 ^+^
	C:K	0.19 ***	−0.07	0.16 ***
	N:P	0.17 ***	−0.09	0.09 *
	N:K	0.20 ***	−0.10	0.21 ***
	P:K	−0.04	−0.07	0.05
	Available N:P	0.00	0.07 ^+^	0.00
	Available N:K	0.13 ***	0.08 **	0.04
	Available P:K	0.14 ***	−0.03	0.07 *
	Plant height	0.08 *	0.03	0.09 *
	Leaf area	0.15 ***	0.00	−0.09
	Stem diameter	0.09 *	0.02	0.06 ^+^
10–20 cm	Longitude	0.17 ***	0.10 *	0.25 ***
	Latitude	0.14 ***	-0.05	0.03
	Elevation	0.19 ***	0.01	0.00
	MAT	0.11 **	0.02	−0.06
	MAP	0.08 *	0.10 ^+^	0.23 ***
	AT2020	0.08 **	0.01	−0.11
	AP2020	0.05	0.19 **	0.26 ***
	EC	0.13 ***	0.02	0.08 *
	pH	0.19 ***	0.01	0.00
	SOC	0.11 **	−0.01	0.09 *
	TN	0.09 *	−0.02	0.04
	TP	−0.02	0.07	0.01
	TK	0.12 ***	0.01	0.05
	NH_4_^+^-N	0.12 ***	0.13 **	0.13 **
	NO_3_^−^-N	0.15 ***	−0.02	0.10 *
	AP	−0.04	0.02	0.04
	AK	0.09 **	0.01	0.15 ***
	C:N	0.03	−0.01	−0.05
	C:P	0.13 ***	0.00	0.03
	C:K	0.11 **	−0.02	0.08 *
	N:P	0.11 **	0.00	−0.03
	N:K	0.09 *	−0.03	0.04
	P:K	0.00	0.05	0.00
	Available N:P	0.08 *	0.07 ^+^	0.00
	Available N:K	0.09 **	0.07 ^+^	0.17 ***
	Available P:K	0.12 **	−0.01	0.17 ***
	Plant height	0.14 ***	0.00	0.18 ***
	Stem diameter	0.10 **	0.06 ^+^	0.06
	Leaf area	0.18 ***	−0.01	−0.07

^+^, *, ** and *** indicates *p* < 0.10, *p* < 0.05, *p* < 0.01 and *p* < 0.001, respectively.

## Data Availability

Not applicable.
